# Supplementary material for: Studying kinematic linkage of finger joints: estimation of kinematics of distal interphalangeal joints during manipulation
Source: PeerJ. 2022 Oct 4;10:e14051. doi: 10.7717/peerj.14051 (PMC9541616; doi:10.7717/peerj.14051)
Supplement: Supplemental Information 1 [file peerj-10-14051-s001.pdf]

Supplemental Figures

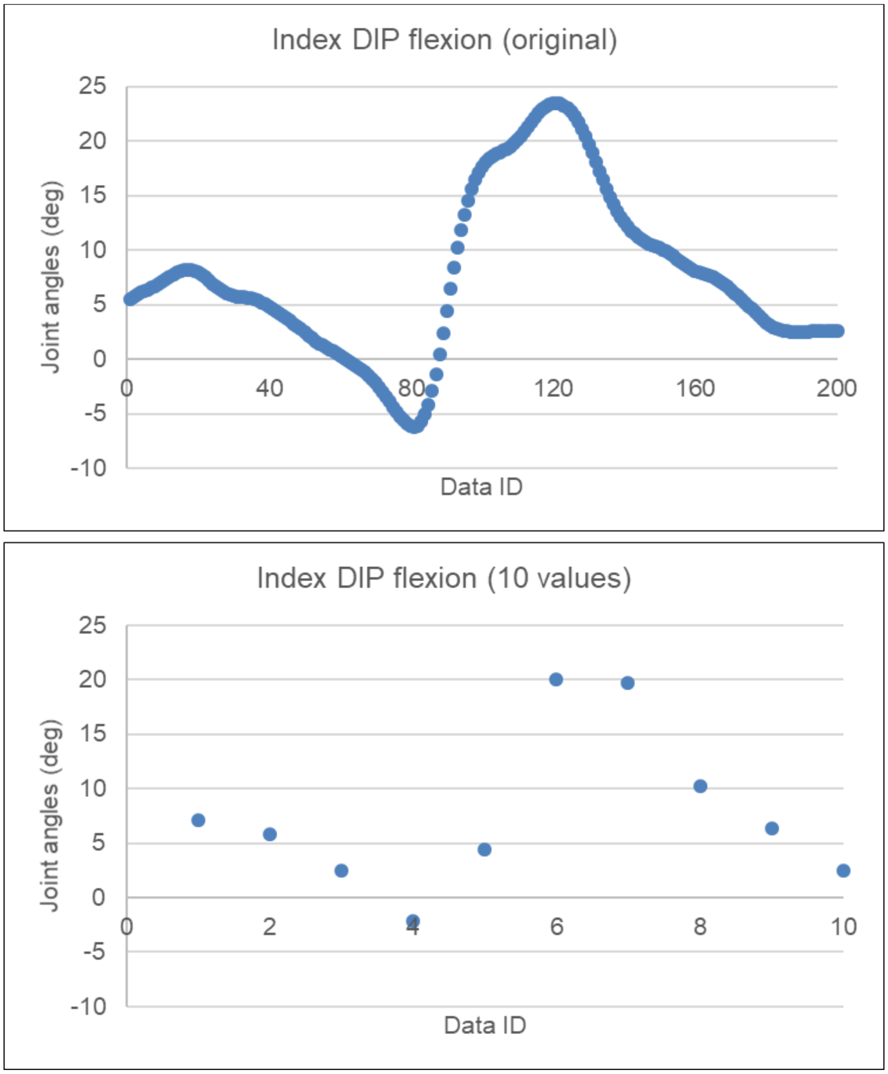

Supp. Fig. 1: Index DIP flexion data during the manipulation phase of task 1 (picking up a coin from a flat surface, and putting it into a purse mounted on a wall). Original collected data (top) and data reduced to 10 samples (bottom).

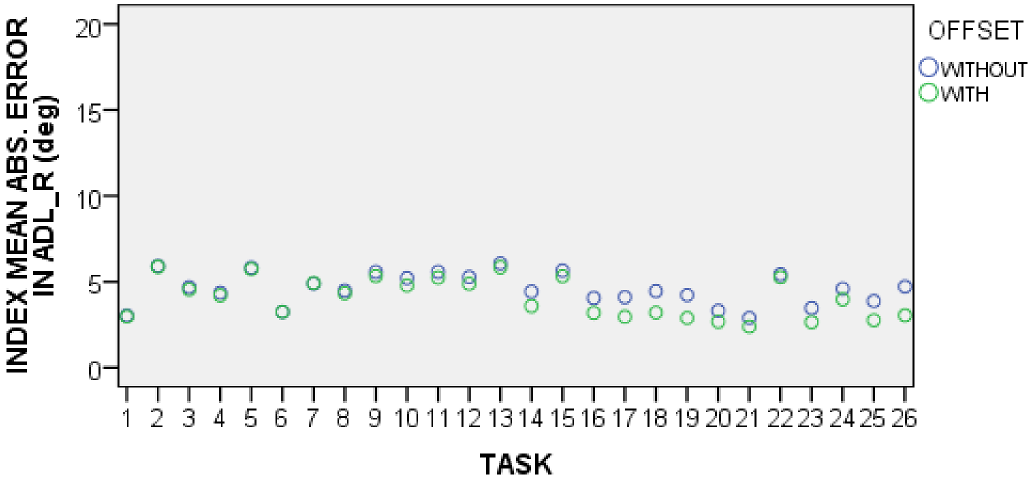

Supp. Fig. 2: Scatter plots of index finger mean absolute error (in degrees) when estimating DIP angles from PIP ones in ADL_M using coefficients obtained when performing regressions with ADL_R data assuming null constant coefficient (without offset) and non-null constant coefficient (with offset). Tasks #1 to #26 (ADLs performance) labeled as in Table 2.

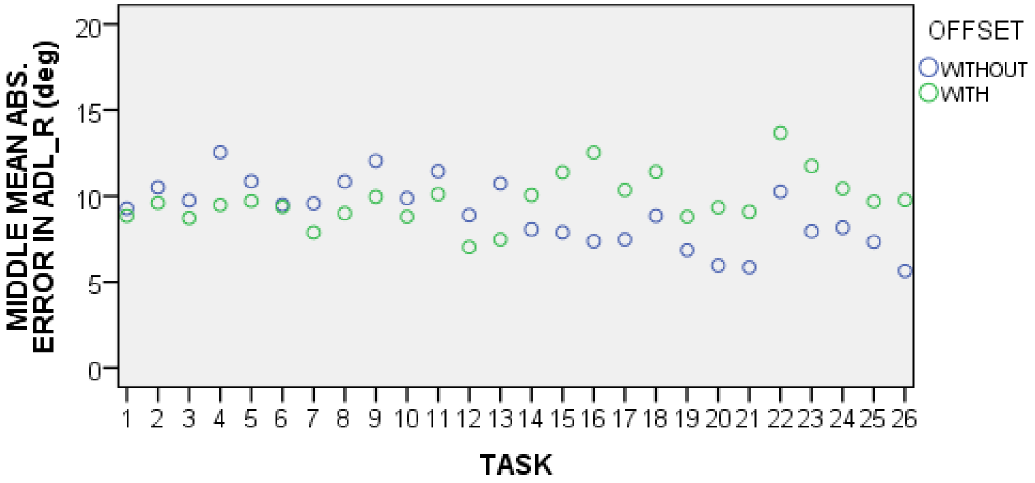

Supp. Fig. 3: Scatter plots of middle finger mean absolute error (in degrees) when estimating DIP angles from PIP ones in ADL_M using coefficients obtained when performing regressions with ADL_R data assuming null constant coefficient (without offset) and non-null constant coefficient (with offset). Tasks #1 to #26 (ADLs performance) labeled as in Table 2.

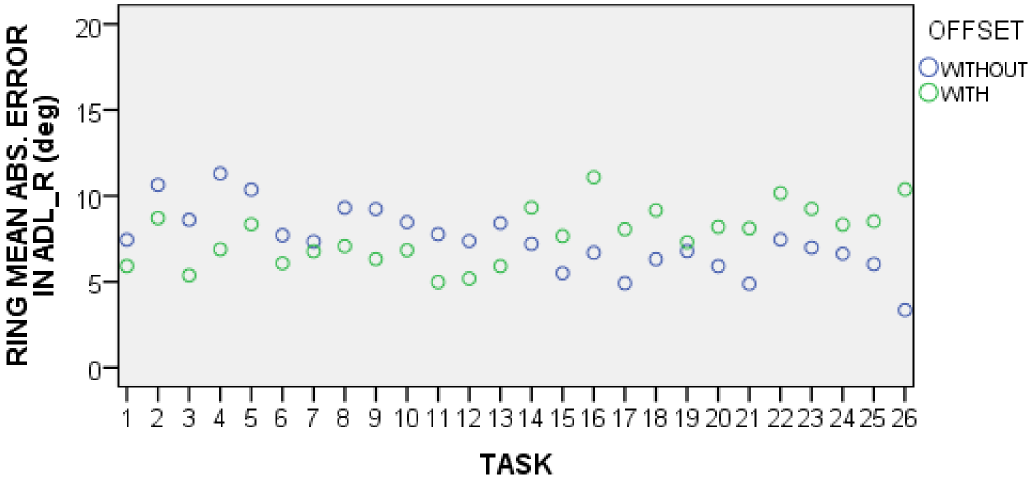

Supp. Fig. 4: Scatter plots of ring finger mean absolute error (in degrees) when estimating DIP angles from PIP ones in ADL_M using coefficients obtained when performing regressions with ADL_R data assuming null constant coefficient (without offset) and non-null constant coefficient (with offset). Tasks #1 to #26 (ADLs performance) labeled as in Table 2.

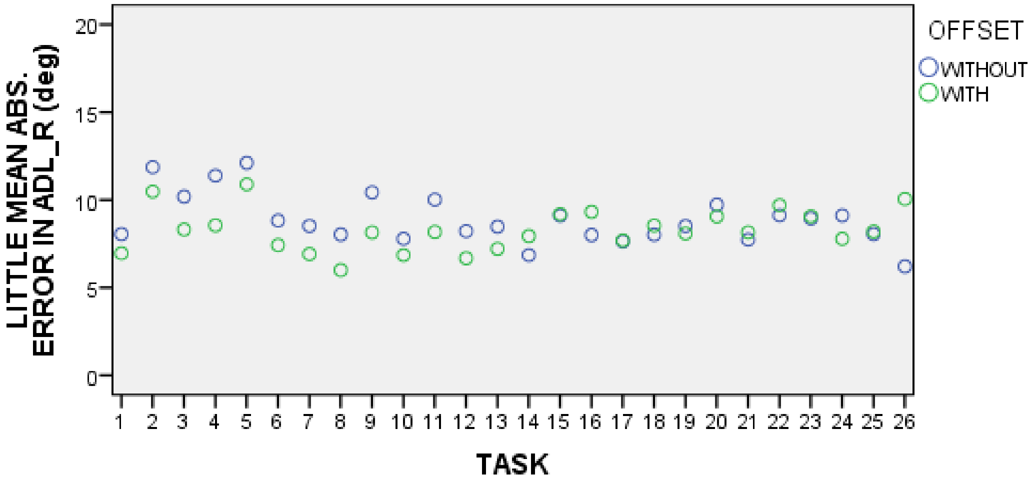

Supp. Fig. 5: Scatter plots of little finger mean absolute error (in degrees) when estimating DIP angles from PIP ones in ADL_M using coefficients obtained when performing regressions with ADL_R data assuming null constant coefficient (without offset) and non-null constant coefficient (with offset). Tasks #1 to #26 (ADLs performance) labeled as in Table 2.

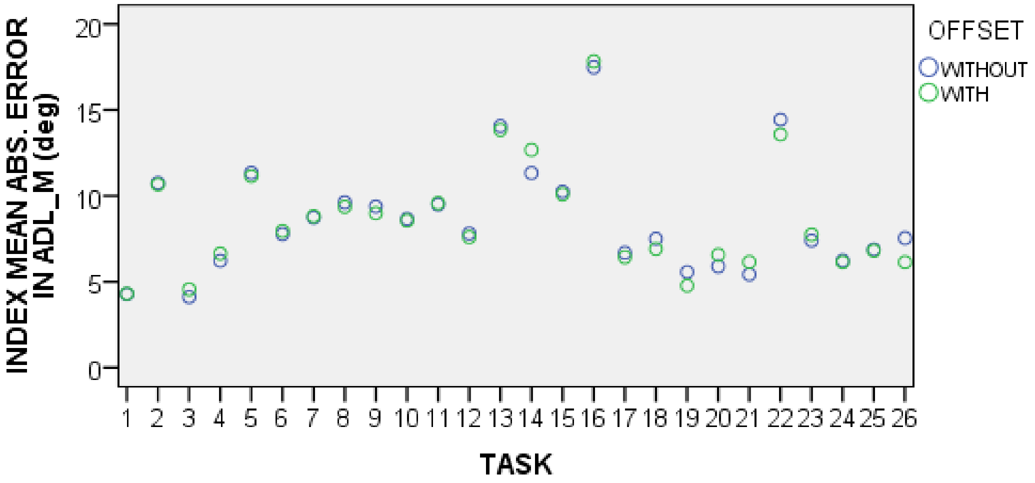

Supp. Fig. 6: Scatter plots of index finger mean absolute error (in degrees) when estimating DIP angles from PIP ones in ADL_M using coefficients obtained when performing regressions with ADL_M data assuming null constant coefficient (without offset) and non-null constant coefficient (with offset). Tasks #1 to #26 (ADLs performance) labeled as in Table 2.

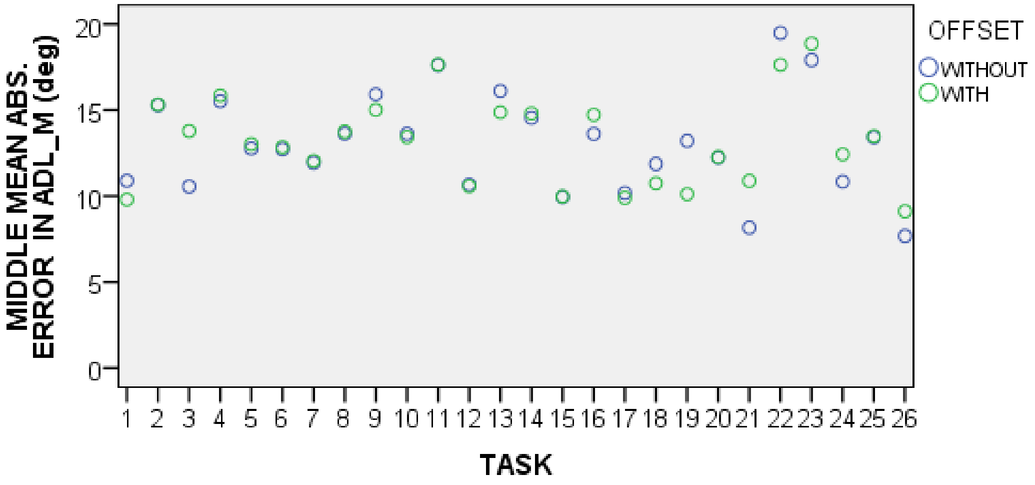

Supp. Fig. 7: Scatter plots of middle finger mean absolute error (in degrees) when estimating DIP angles from PIP ones in ADL_M using coefficients obtained when performing regressions with ADL_M data assuming null constant coefficient (without offset) and non-null constant coefficient (with offset). Tasks #1 to #26 (ADLs performance) labeled as in Table 2.

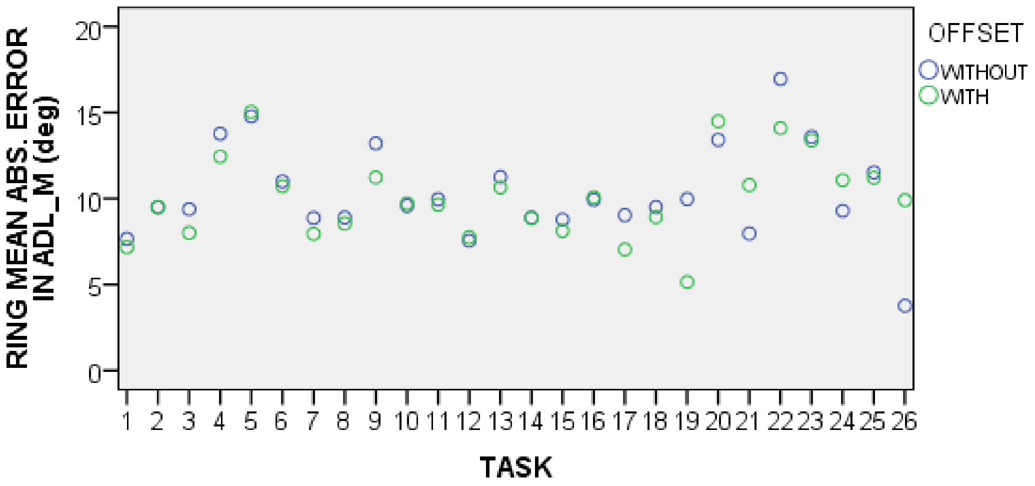

Supp. Fig. 8: Scatter plots of ring finger mean absolute error (in degrees) when estimating DIP angles from PIP ones in ADL_M using coefficients obtained when performing regressions with ADL_M data assuming null constant coefficient (without offset) and non-null constant coefficient (with offset). Tasks #1 to #26 (ADLs performance) labeled as in Table 2.

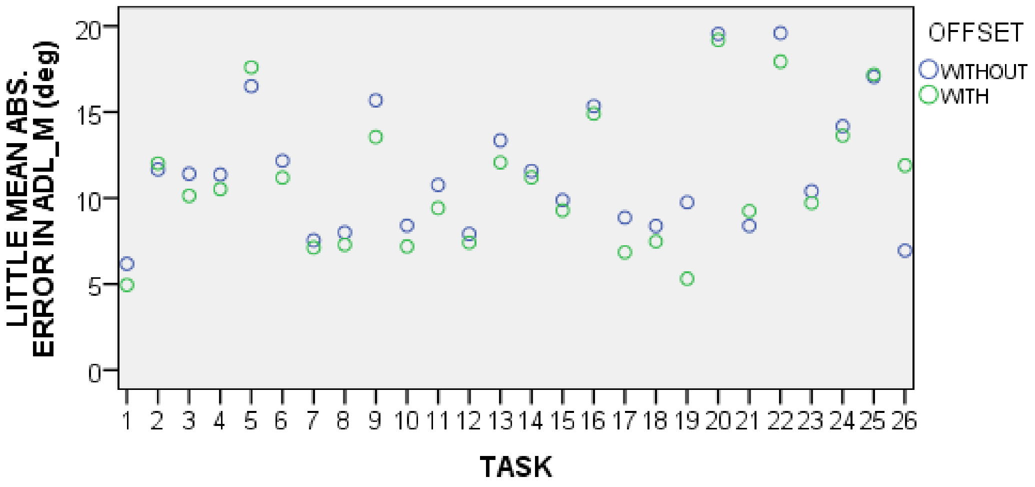

Supp. Fig. 9: Scatter plots of little finger mean absolute error (in degrees) when estimating DIP angles from PIP ones in ADL_M using coefficients obtained when performing regressions with ADL_M data assuming null constant coefficient (without offset) and non-null constant coefficient (with offset). Tasks #1 to #26 (ADLs performance) labeled as in Table 2.

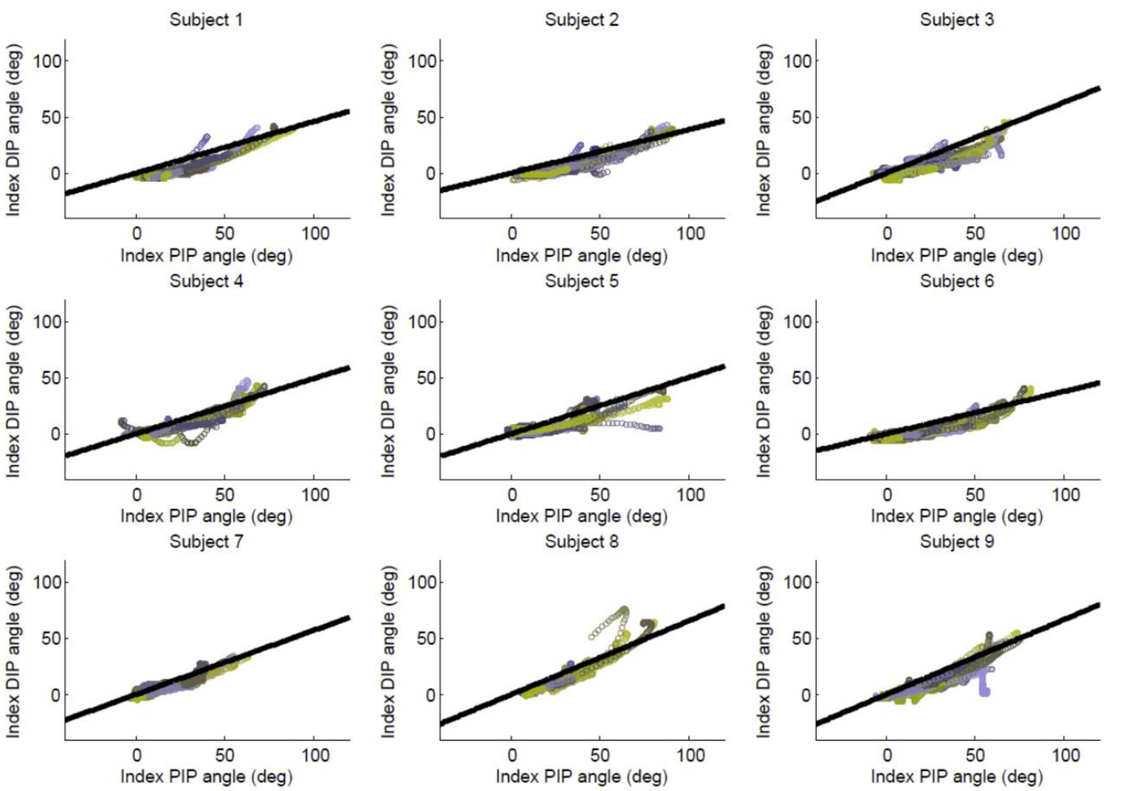

Supp. Fig. 10: Scatter plots of index finger PIP and DIP angles recorded (in degrees) during ADL_R, for each subject. Each task data plotted with a different colour. Regression line of each subject's FMT data plotted in black.

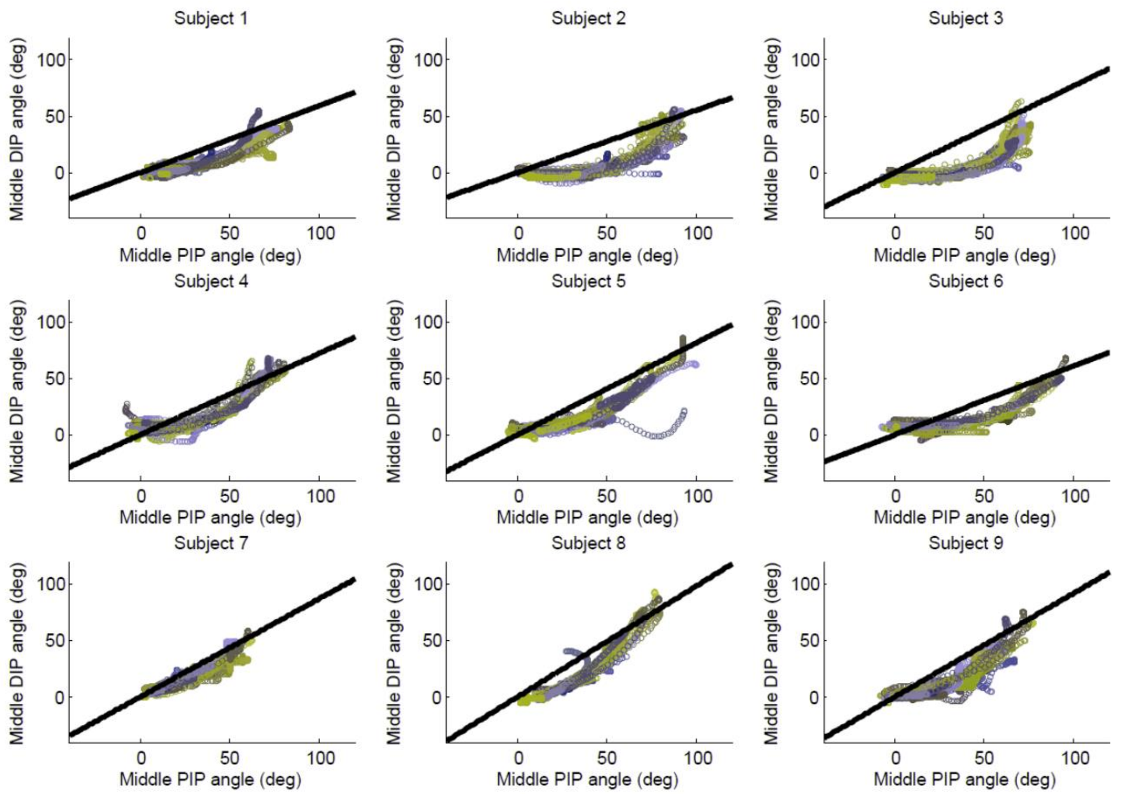

Supp. Fig. 11: Scatter plots of middle finger PIP and DIP angles recorded (in degrees) during ADL_R, for each subject. Each task data plotted with a different colour. Regression line of each subject's FMT data plotted in black

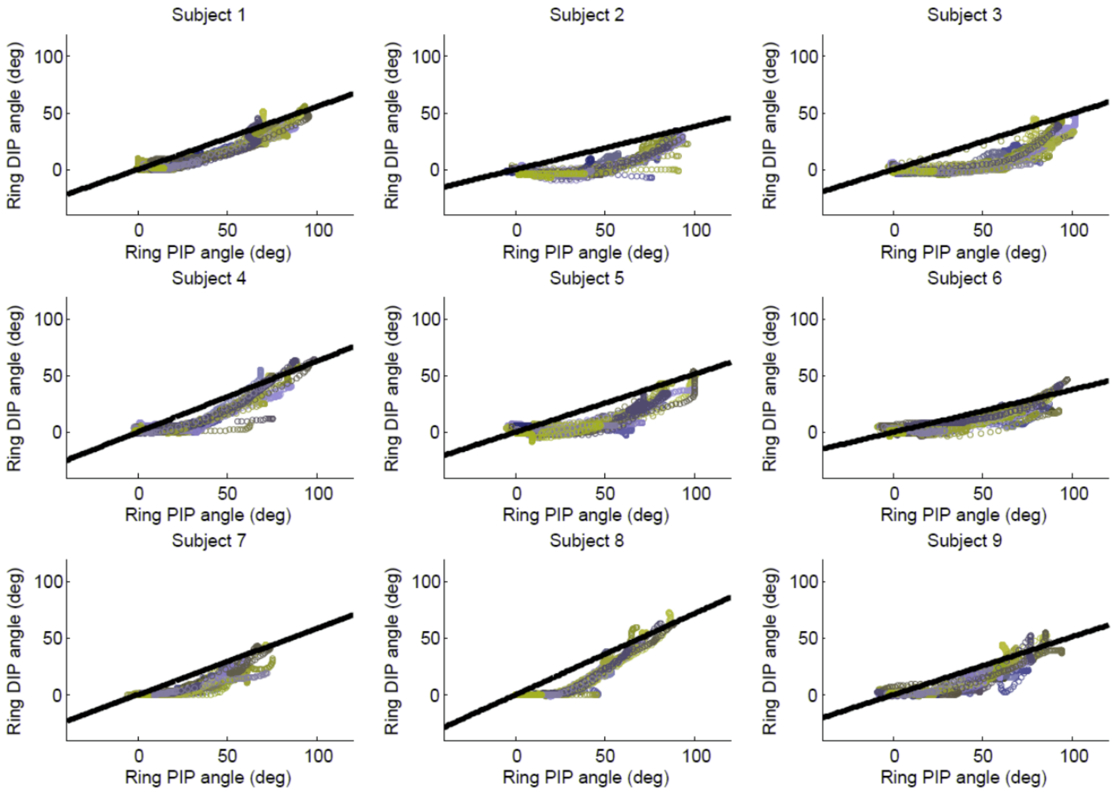

Supp. Fig. 12: Scatter plots of ring finger PIP and DIP angles recorded (in degrees) during ADL_R, for each subject. Each task data plotted with a different colour. Regression line of each subject's FMT data plotted in black.

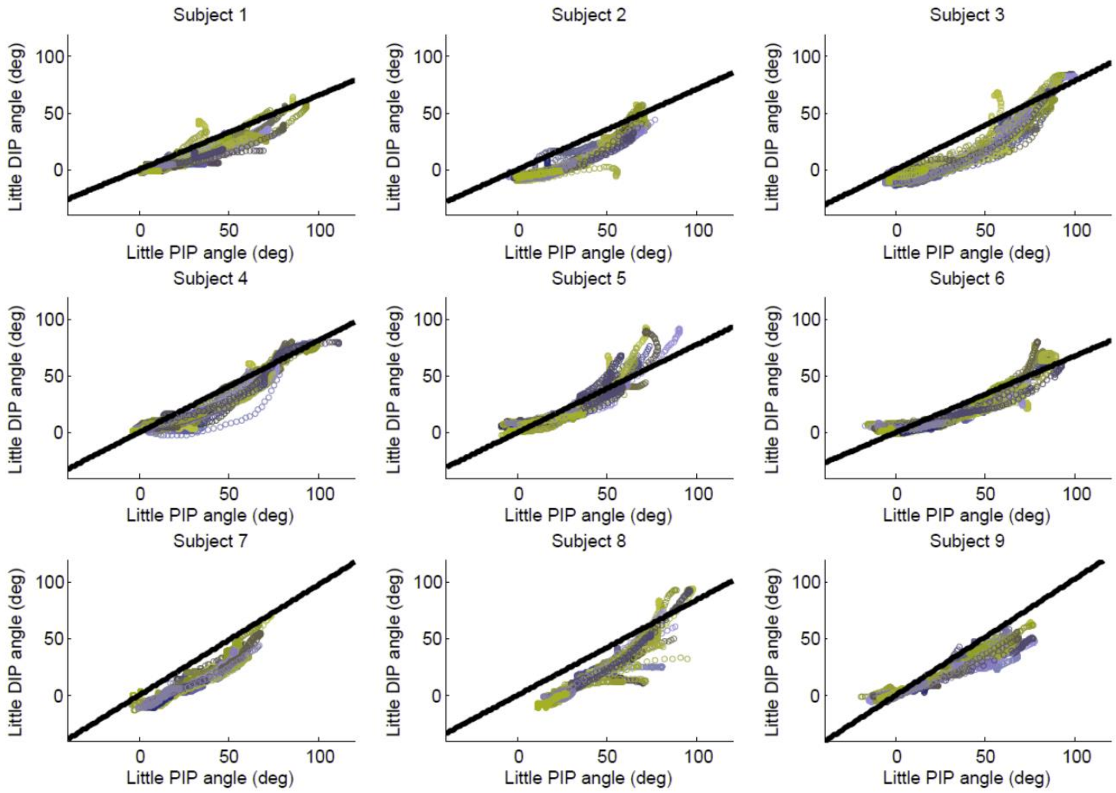

Supp. Fig. 13: Scatter plots of little finger PIP and DIP angles recorded (in degrees) during ADL_R, for each subject. Each task data plotted with a different colour. Regression line of each subject's FMT data plotted in black.

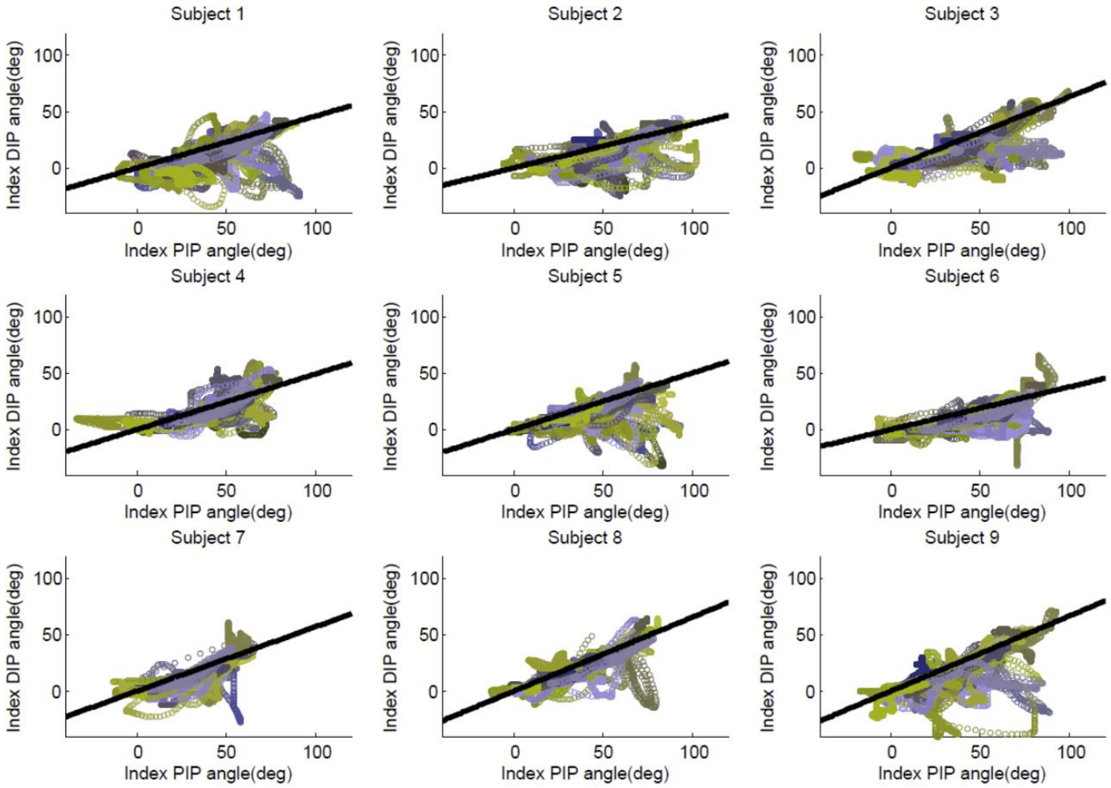

Supp. Fig. 14: Scatter plots of index finger PIP and DIP angles recorded (in degrees) during ADL_M, for each subject. Each task data plotted with a different colour. Regression line of each subject's FMT data plotted in black.

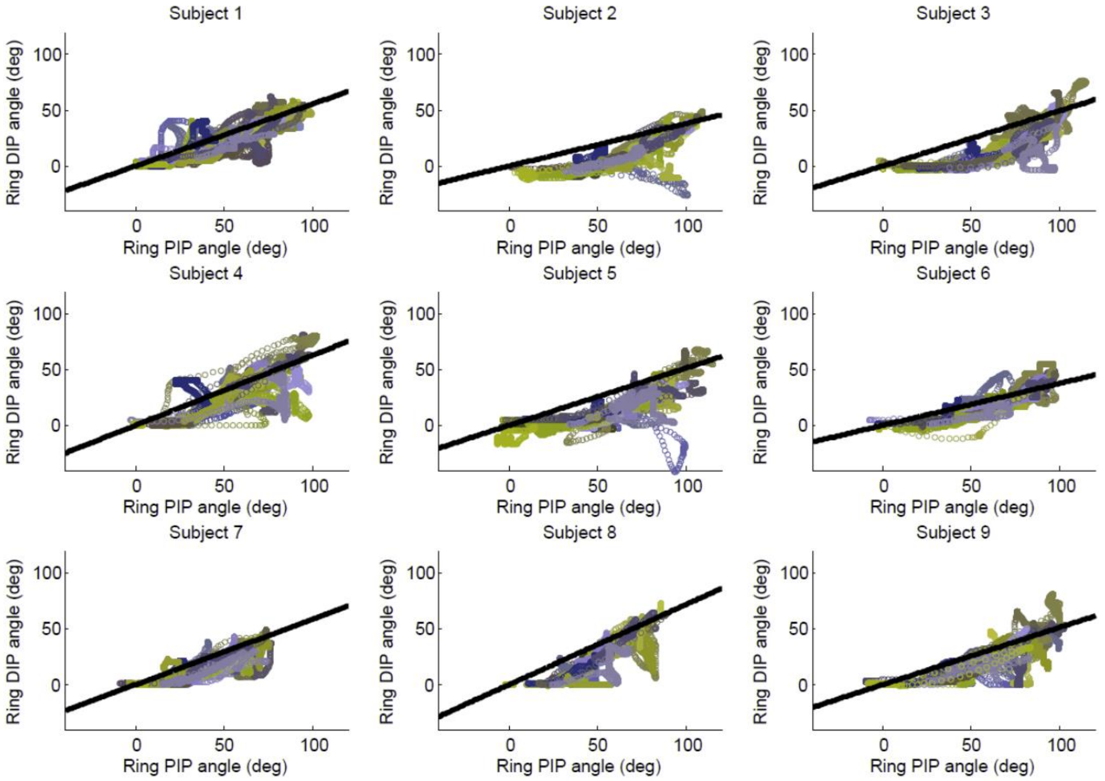

Supp. Fig. 15: Scatter plots of middle finger PIP and DIP angles recorded (in degrees) during ADL_M, for each subject. Each task data plotted with a different colour. Regression line of each subject's FMT data plotted in black.

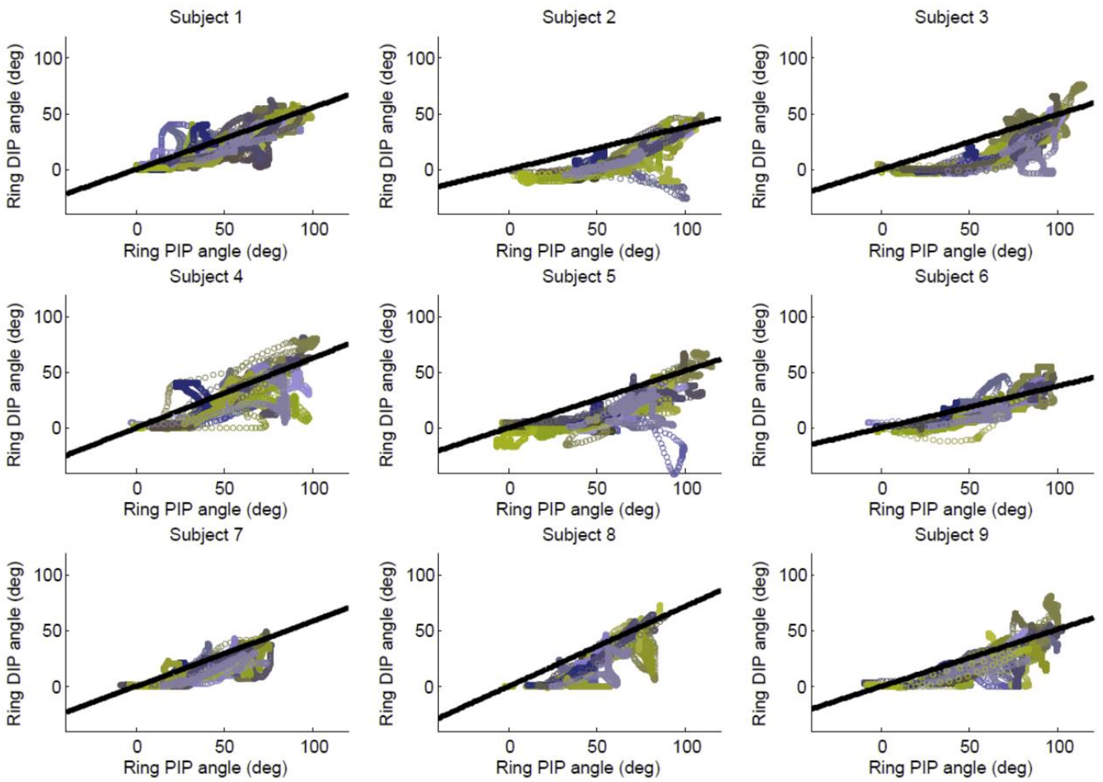

Supp. Fig. 16: Scatter plots of ring finger PIP and DIP angles recorded (in degrees) during ADL_M, for each subject. Each task data plotted with a different colour. Regression line of each subject's FMT data plotted in black.

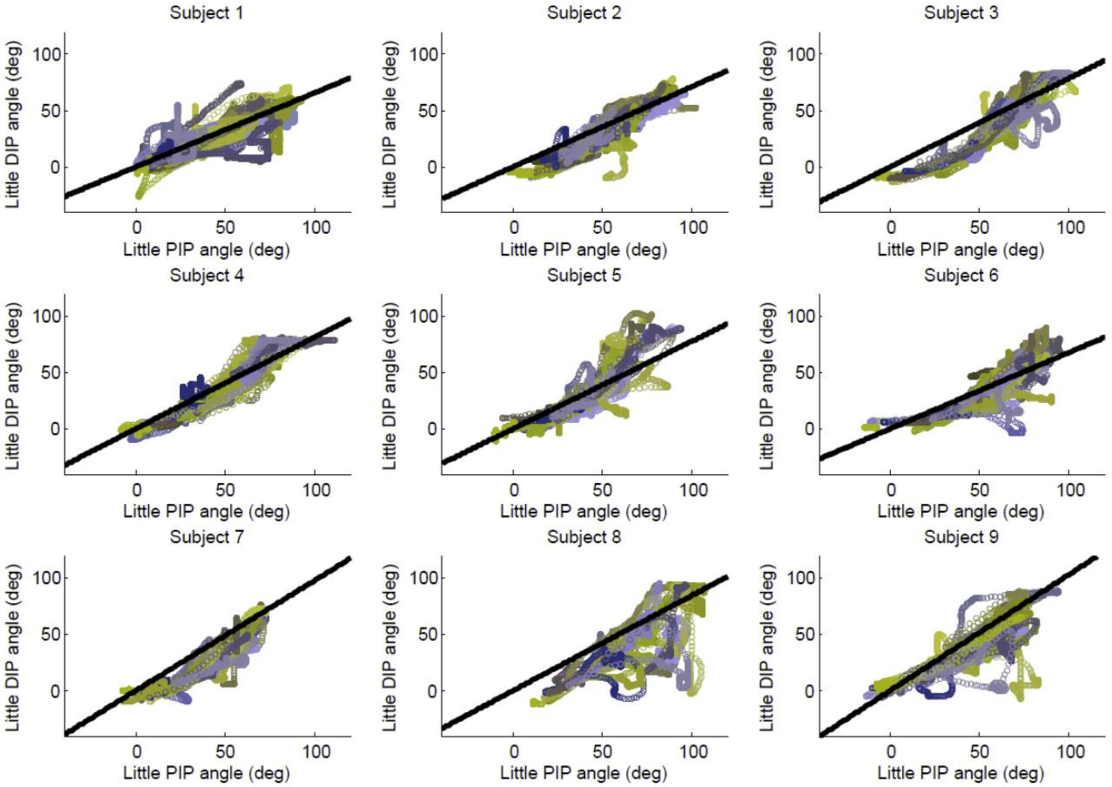

Supp. Fig. 17: Scatter plots of little finger PIP and DIP angles recorded (in degrees) during ADL_M, for each subject. Each task data plotted with a different colour. Regression line of each subject's FMT data plotted in black.

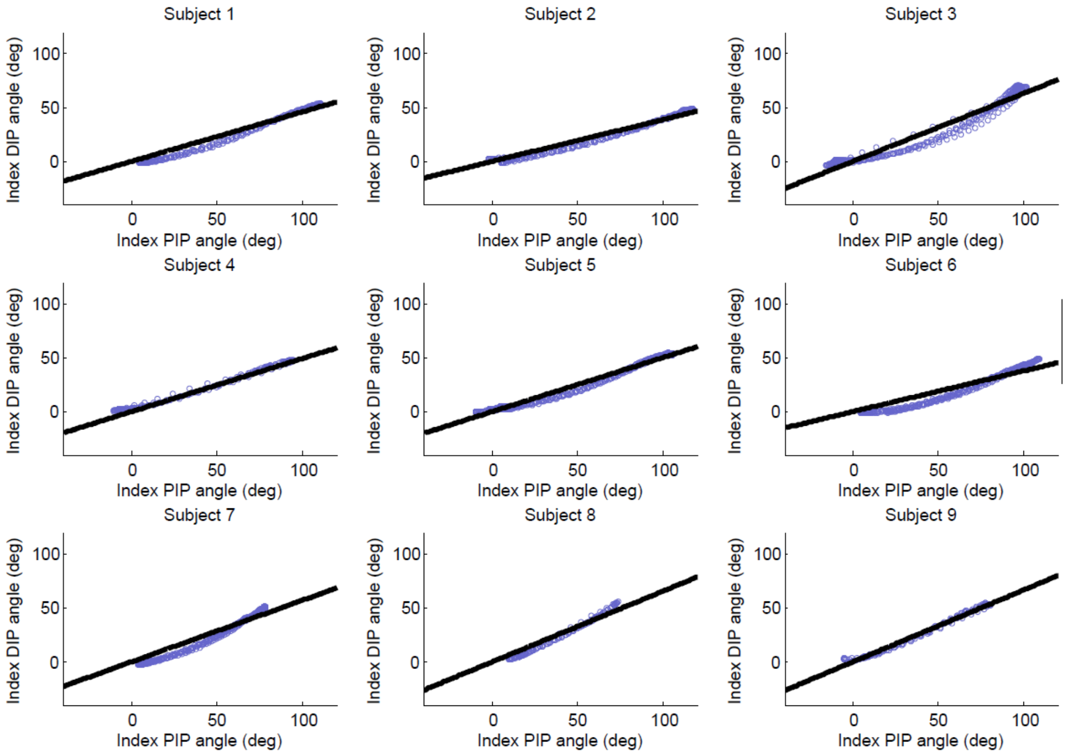

Supp. Fig. 18: Scatter plots of index finger PIP and DIP angles recorded (in degrees) during FMT, for each subject. Regression line of each subject's data plotted in black.

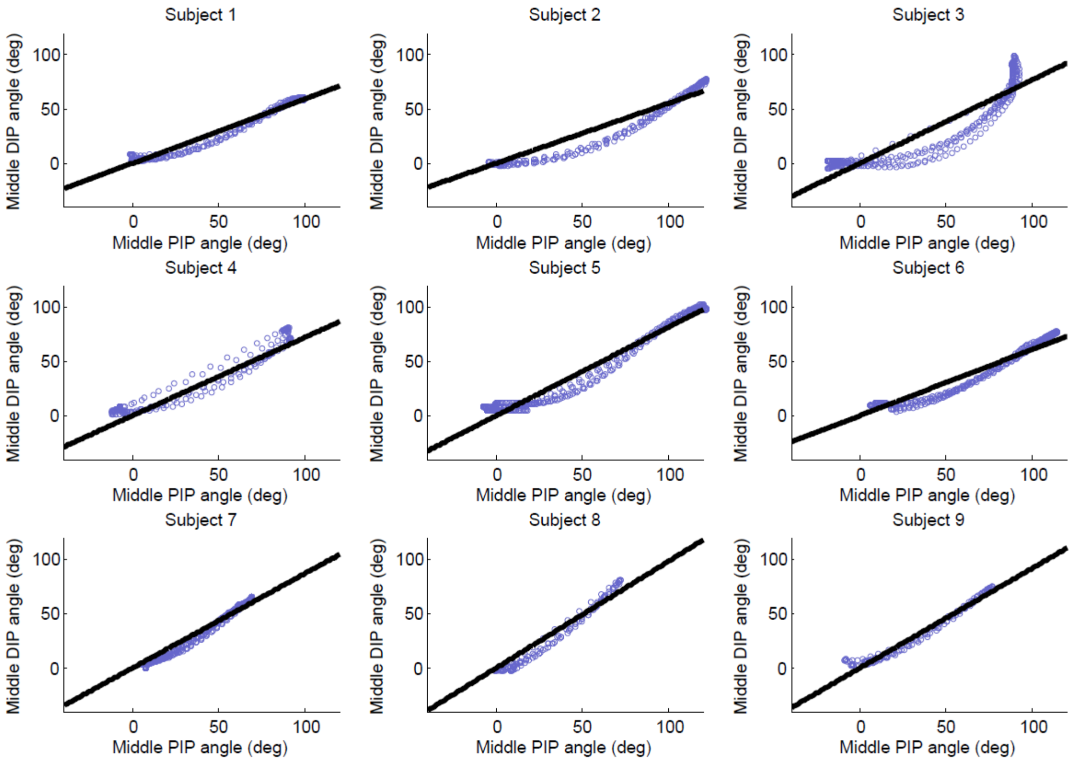

Supp. Fig. 19: Scatter plots of middle finger PIP and DIP angles recorded (in degrees) during FMT, for each subject. Regression line of each subject's data plotted in black.

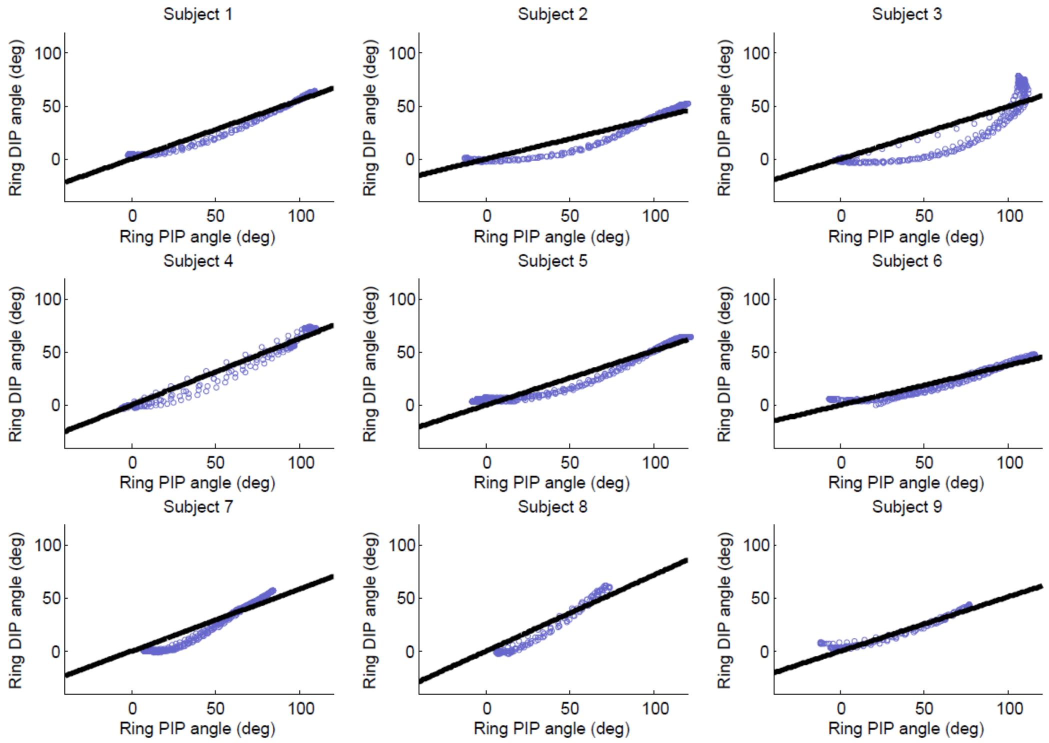

Supp. Fig. 20: Scatter plots of ring finger PIP and DIP angles recorded (in degrees) during FMT, for each subject. Regression line of each subject's data plotted in black.

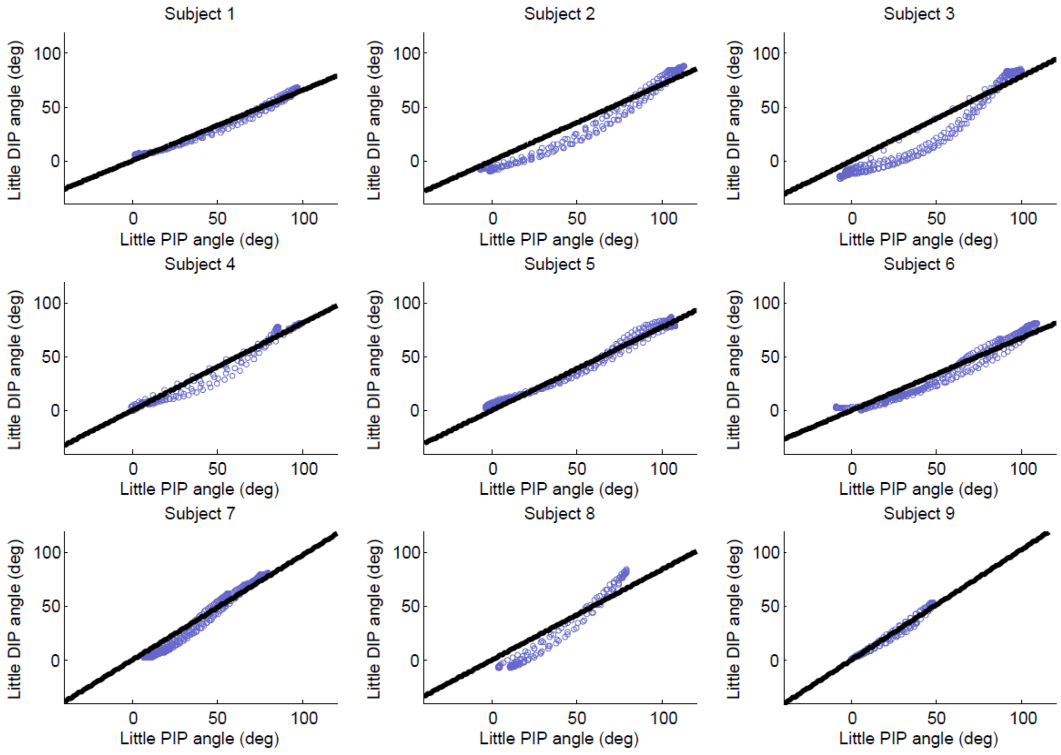

Supp. Fig. 21: Scatter plots of little finger PIP and DIP angles recorded (in degrees) during FMT, for each subject. Regression line of each subject's data plotted in black.

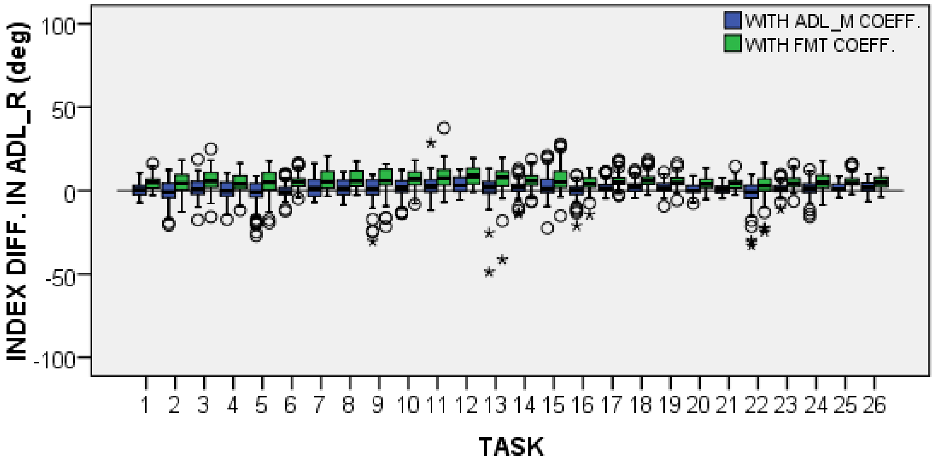

Supp. Fig. 22: Box and whiskers plots of error (in degrees) obtained in each task during ADL_R when estimating the index DIP angle from the PIP one using the coefficients obtained during FMT (in green) and during ADL_M (in blue). Tasks #1 to #26 (ADLs performance) labeled as in Table 2.Outliers marked with a circle, extreme outliers marked with an asterisk.

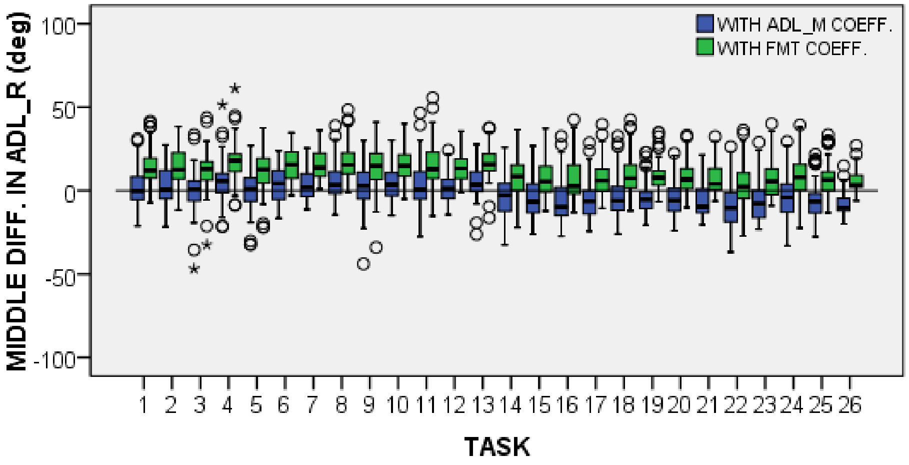

Supp. Fig. 23: Box and whiskers plots of error (in degrees) obtained in each task during ADL_R when estimating the middle DIP angle from the PIP one using the coefficients obtained during FMT (in green) and during ADL_M (in blue). Tasks #1 to #26 (ADLs performance) labeled as in Table 2. Outliers marked with a circle, extreme outliers marked with an asterisk.

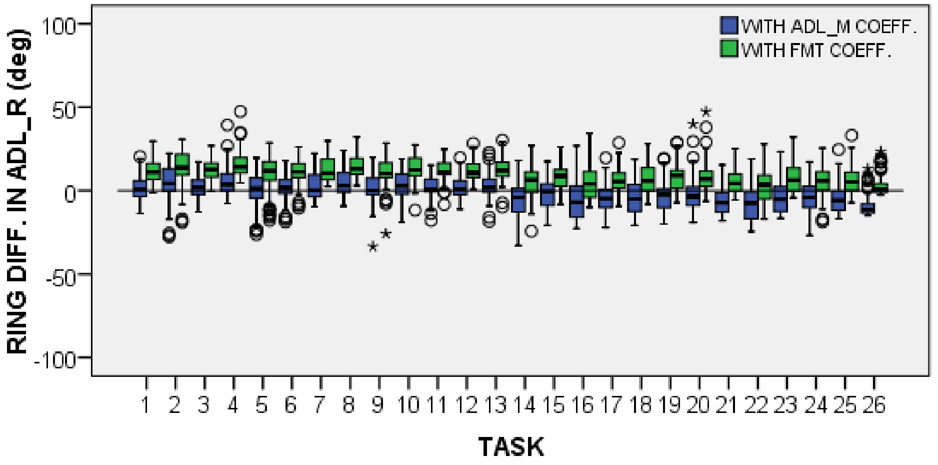

Supp. Fig. 24: Box and whiskers plots of error (in degrees) obtained in each task during ADL_R when estimating the ring DIP angle from the PIP one using the coefficients obtained during FMT (in green) and during ADL_M (in blue). Tasks #1 to #26 (ADLs performance) labeled as in Table 2. Outliers marked with a circle, extreme outliers marked with an asterisk.

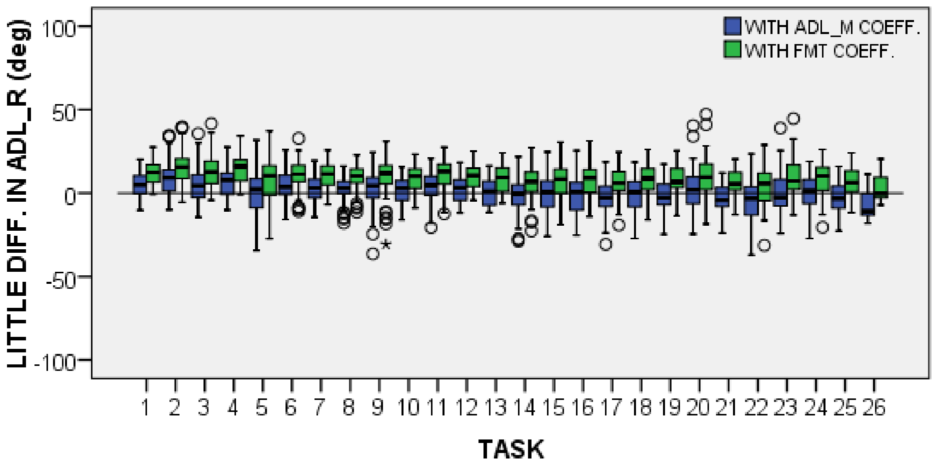

Supp. Fig. 25: Box and whiskers plots of error (in degrees) obtained in each task during ADL_R when estimating the little DIP angle from the PIP one using the coefficients obtained during FMT (in green) and during ADL_M (in blue). Tasks #1 to #26 (ADLs performance) labeled as in Table 2. Outliers marked with a circle, extreme outliers marked with an asterisk.

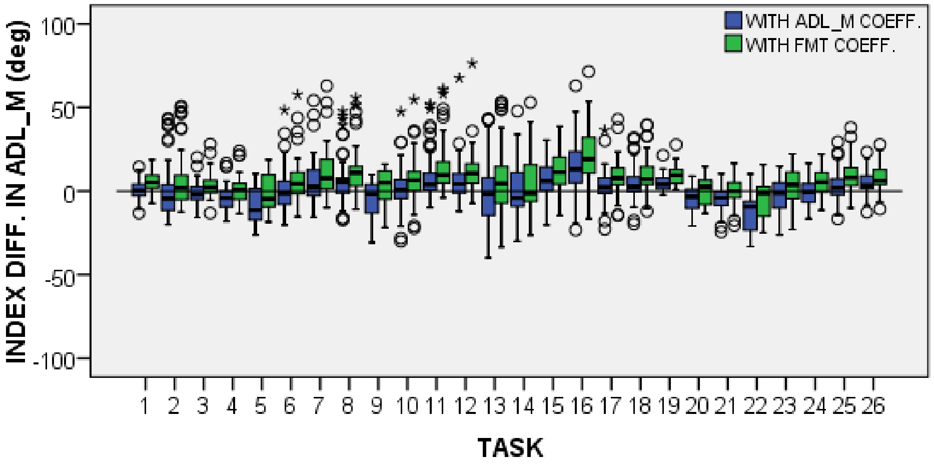

Supp. Fig. 26: Box and whiskers plots of error (in degrees) obtained in each task during ADL_M when estimating the index DIP angle from the PIP one using the coefficients obtained during FMT (in green) and during ADL_M (in blue). Tasks #1 to #26 (ADLs performance) labeled as in Table 2. Outliers marked with a circle, extreme outliers marked with an asterisk.

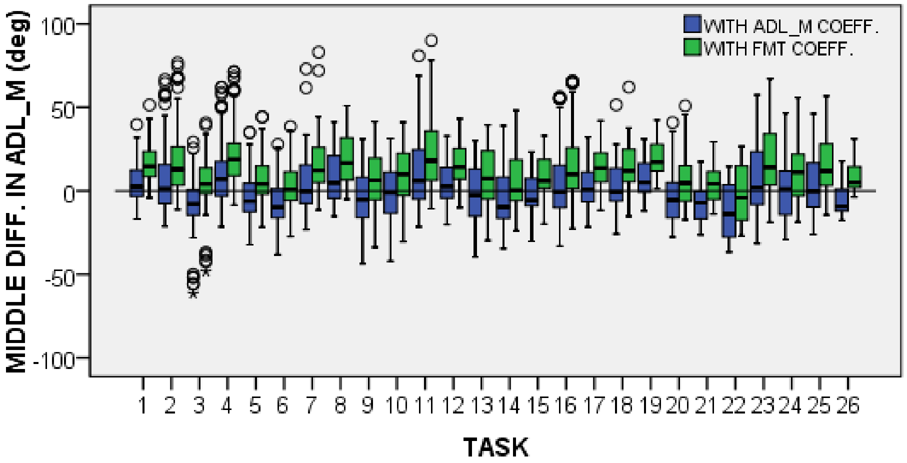

Supp. Fig. 27: Box and whiskers plots of error (in degrees) obtained in each task during ADL_M when estimating the middle DIP angle from the PIP one using the coefficients obtained during FMT (in green) and during ADL_M (in blue). Tasks #1 to #26 (ADLs performance) labeled as in Table 2. Outliers marked with a circle, extreme outliers marked with an asterisk.

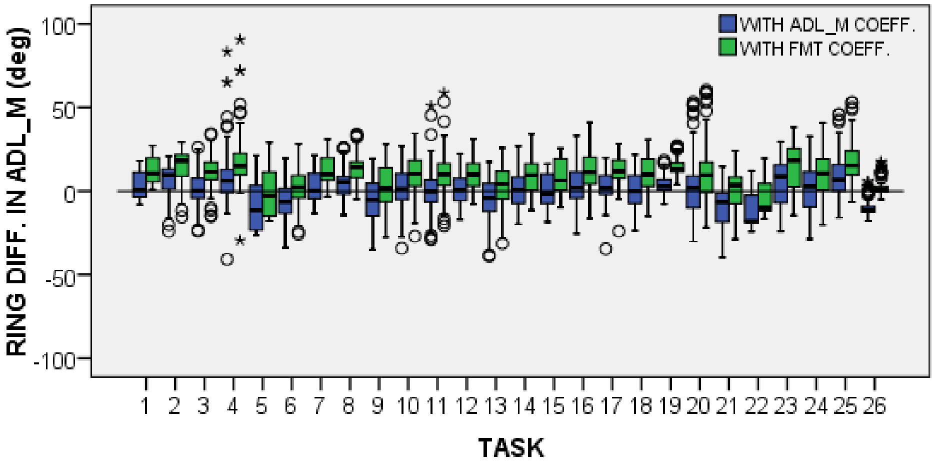

Supp. Fig. 28: Box and whiskers plots of error (in degrees) obtained in each task during ADL_M when estimating the ring DIP angle from the PIP one using the coefficients obtained during FMT (in green) and during ADL_M (in blue). Tasks #1 to #26 (ADLs performance) labeled as in Table 2. Outliers marked with a circle, extreme outliers marked with an asterisk.

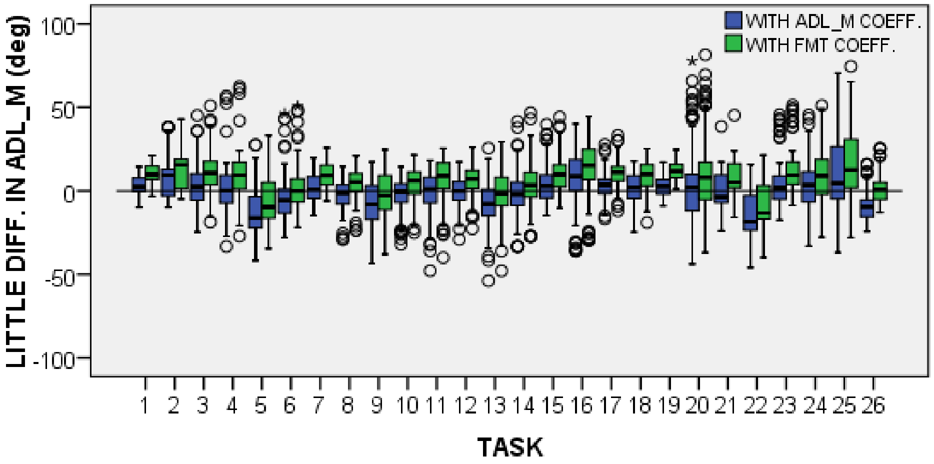

Supp. Fig. 29: Box and whiskers plots of error (in degrees) obtained in each task during ADL_M when estimating the little DIP angle from the PIP one using the coefficients obtained during FMT (in green) and during ADL_M (in blue). Tasks #1 to #26 (ADLs performance) labeled as in Table 2. Outliers marked with a circle, extreme outliers marked with an asterisk.

Supplemental Tables

	Index		Middle		Ring		Little	
Task	p	Partial Eta Squared	p	Partial Eta Squared	p	Partial Eta Squared	p	Partial Eta Squared
1	0.000	0.361	0.000	0.268	0.000	0.519	0.000	0.529
2	0.093	0.031	0.000	0.390	0.000	0.623	0.000	0.646
3	0.000	0.294	0.000	0.274	0.000	0.721	0.000	0.490
4	0.001	0.120	0.000	0.568	0.000	0.908	0.000	0.613
5	0.008	0.077	0.000	0.259	0.000	0.295	0.002	0.104
6	0.000	0.316	0.000	0.319	0.000	0.632	0.000	0.587
7	0.000	0.226	0.000	0.633	0.000	0.583	0.000	0.321
8	0.000	0.357	0.000	0.577	0.000	0.851	0.000	0.612
9	0.000	0.304	0.000	0.434	0.000	0.621	0.000	0.485
10	0.000	0.435	0.000	0.475	0.000	0.650	0.000	0.262
11	0.000	0.499	0.000	0.421	0.000	0.630	0.000	0.390
12	0.000	0.654	0.000	0.500	0.000	0.624	0.000	0.301
13	0.000	0.380	0.000	0.851	0.000	0.702	0.000	0.139
14	0.000	0.421	0.221	0.017	0.974	0.000	0.177	0.020
15	0.000	0.420	0.342	0.010	0.036	0.049	0.017	0.062
16	0.000	0.250	0.066	0.037	0.042	0.046	0.140	0.024
17	0.000	0.719	0.812	0.001	0.447	0.007	0.098	0.031
18	0.000	0.811	0.880	0.000	0.959	0.000	0.051	0.042
19	0.000	0.768	0.435	0.007	0.085	0.033	0.021	0.058
20	0.000	0.428	0.865	0.000	0.246	0.015	0.000	0.140
21	0.000	0.420	0.494	0.005	0.096	0.031	0.385	0.008
22	0.215	0.017	0.002	0.106	0.032	0.051	0.001	0.106
23	0.000	0.453	0.177	0.020	0.975	0.000	0.079	0.034
24	0.000	0.333	0.465	0.006	0.711	0.002	0.000	0.181
25	0.000	0.692	0.600	0.003	0.447	0.007	0.235	0.016
26	0.000	0.818	0.001	0.112	0.000	0.384	0.000	0.131

Supp. Table 1: Observed p value and partial eta squared in the repeated measures ANOVAs performed with the mean error when estimating DIP angles from PIP ones in ADL_R, classified by fingers.

	Index		Middle		Ring		Little	
Task	p	Partial Eta Squared	p	Partial Eta Squared	p	Partial Eta Squared	p	Partial Eta Squared
1	0.000	0.203	0.000	0.411	0.000	0.495	0.000	0.727
2	0.048	0.043	0.000	0.160	0.000	0.592	0.000	0.252
3	0.430	0.007	0.131	0.025	0.000	0.314	0.000	0.199
4	0.111	0.028	0.000	0.420	0.000	0.616	0.002	0.098
5	0.225	0.016	0.520	0.005	0.000	0.158	0.000	0.268
6	0.133	0.025	0.024	0.056	0.060	0.039	0.013	0.067
7	0.000	0.262	0.000	0.253	0.000	0.369	0.000	0.226
8	0.000	0.264	0.000	0.540	0.000	0.455	0.012	0.069
9	0.482	0.006	0.878	0.000	0.935	0.000	0.169	0.021
10	0.006	0.082	0.081	0.034	0.000	0.196	0.009	0.074
11	0.000	0.619	0.000	0.338	0.000	0.172	0.000	0.163
12	0.000	0.452	0.000	0.356	0.000	0.187	0.000	0.141
13	0.948	0.000	0.217	0.017	0.997	0.000	0.171	0.021
14	0.082	0.034	0.163	0.022	0.029	0.052	0.879	0.000
15	0.000	0.573	0.103	0.030	0.004	0.090	0.000	0.313
16	0.000	0.519	0.005	0.086	0.000	0.218	0.000	0.394
17	0.000	0.389	0.000	0.244	0.000	0.575	0.000	0.474
18	0.000	0.456	0.000	0.245	0.000	0.175	0.000	0.231
19	0.000	0.888	0.000	0.807	0.000	0.910	0.000	0.688
20	0.197	0.019	0.678	0.002	0.022	0.057	0.034	0.050
21	0.004	0.092	0.209	0.018	0.050	0.042	0.092	0.032
22	0.000	0.531	0.000	0.133	0.000	0.259	0.000	0.196
23	0.043	0.045	0.005	0.084	0.000	0.162	0.000	0.227
24	0.024	0.056	0.107	0.029	0.006	0.081	0.002	0.104
25	0.000	0.304	0.001	0.125	0.000	0.696	0.000	0.174
26	0.000	0.633	0.296	0.012	0.000	0.287	0.000	0.252

Supp. Table 2: Observed p value and partial eta squared in the repeated measures ANOVAs performed with the mean error when estimating DIP angles from PIP ones in ADL_M, classified by fingers.
